# Prognostic value of neutrophil-to-lymphocyte ratio change in patients with locally advanced non-small cell lung cancer treated with thoracic radiotherapy

**DOI:** 10.1038/s41598-024-62662-3

**Published:** 2024-05-25

**Authors:** Xiaoming Yin, Haijun Chen, Yunchuan Sun, Li Xiao, Hongling Lu, Wei Guo, Hongjuan Yang, Jianxi Zhou, Kui Fan, Wei Liang

**Affiliations:** 1 Department of Radiation Oncology, Cangzhou Hospital of Integrated Traditional Chinese and Western Medicine of Hebei Province, Affiliated Hospital of Hebei Medical University, No. 31, Huanghe West Road, Cangzhou, 061000 Hebei China; 2 Department of Anesthesiology, Cangzhou Central Hospital, Cangzhou, 061000 Hebei China

**Keywords:** Non-small-cell lung cancer, Radiotherapy

## Abstract

In prior investigations, a correlation was established between patient outcomes in locally advanced non-small cell lung cancer (LA-NSCLC) following thoracic irradiation and parameters, such as pre/post-treatment neutrophil-to-lymphocyte ratio (NLR) and NLR change (ΔNLR). However, these parameters could potentially be influenced by radiation-related variables, such as gross tumor volume (GTV). The primary aim of this study was to elucidate the factors impacting post-treatment NLR and ΔNLR and to further assess their prognostic relevance. In this retrospective study, a cohort of 188 LA-NSCLC patients who underwent thoracic radiation between 2012 and 2017 was assessed. The calculation of pre/post-treatment NLR involved the use of absolute neutrophil and lymphocyte counts. ΔNLR was defined as the difference between post- and pre-treatment NLR values. To assess the relationships between various variables and overall survival (OS), local progression-free survival (LPFS), and distant metastasis-free survival (DMFS), the Kaplan–Meier technique and Cox proportional hazards regression were employed. Additionally, Spearman’s rank correlation analysis was carried out to investigate correlations between the variables. The analysis revealed that both post-treatment NLR (r = 0.315, *P* < 0.001) and ΔNLR (r = 0.156, *P* = 0.032) were associated with GTV. However, OS, LPFS, and DMFS were not independently correlated with pre/post-treatment NLR. ΔNLR, on the other hand, exhibited independent associations with OS and DMFS (HR = 1.054, *P* = 0.020, and *P* = 0.046, respectively). Elevated ΔNLR values were linked to poorer OS (*P* = 0.023) and DMFS (*P* = 0.018) in the Kaplan–Meier analysis. Furthermore, when stratifying by GTV, a higher ΔNLR remained to be associated with worse OS and DMFS (*P* = 0.047 and *P* = 0.035, respectively) in the GTV ≤ 67.41 cm^3^ group, and in the GTV > 67.41 cm^3^ group (*P* = 0.028 and *P* = 0.042, respectively), highlighting ΔNLR as the sole independent predictive factor for survival and metastasis, irrespective of GTV.

## Introduction

Patients with unresectable, locally advanced non-small cell lung cancer (LA-NSCLC) significantly benefit from immunomaintenance treatment after chemoradiotherapy^1–3^. The effects of radiation on the body’s inflammatory state have noticeably attracted scholars’ attention because immunotherapy has been used more frequently as it may alter its effectiveness^4^. As one of the important markers of inflammation, neutrophil-to-lymphocyte ratio (NLR) has been widely studied, and it has been proven to have a predictive value for the prognosis^5–8^. As the immune state is not static and can be changed by radiotherapy and chemotherapy, the dynamic changes of NLR may provide more information compared to a single time-point NLR. However, few studies have concentrated on the impact of dynamic changes of NLR on prognosis. Besides, the prognostic significance of NLR remains controversial. Research has shown that higher pre-treatment NLR, post-treatment NLR, and NLR change (ΔNLR) were independent predictors of poor survival^9^. However, another study revealed that ΔNLR was correlated with overall survival (OS) and progression-free survival (PFS). While post-treatment NLR was solely a standalone predictor of PFS, baseline NLR was not associated with prognosis^10^. Therefore, the predictive significance of the dynamic change of NLR on the prognosis still needs further investigation. It is noteworthy that the abovementioned studies did not profoundly assess the influential factors of post-treatment NLR and ΔNLR. For instance, gross tumor volume (GTV) may affect post-treatment NLR and ΔNLR, and a larger baseline GTV was reported to be related to worse OS^11^. Further research is required to determine whether post-treatment NLR and ΔNLR are independent prognostic variables. The present research aimed to assess the influential factors of post-treatment NLR and ΔNLR and further explore their prognostic significance.

## Methods

### Patients

The study enrolled 188 patients with unresectable LA-NSCLC, including histologically-confirmed stage III, who were treated with radiotherapy at our hospital between 2012 and 2017. Prior to commencement, approval was granted by the Medical Ethics Committee of Cangzhou Hospital of Integrated Traditional Chinese and Western Medicine of Hebei Province (China). All experiments were performed in accordance with relevant guidelines and regulations. In addition, informed consent was obtained from participants and/or their legal guardians. The inclusion criteria were summarized as follows: age > 18 years, patients with non-small cell lung cancer (including squamous cell carcinoma, adenocarcinoma, or large cell carcinoma), patients with disease stage IIIA, IIIB, or IIIC (according to the eighth edition of the American Joint Committee on Cancer), and patients who had received conventionally fractionated radiotherapy and completed the course of treatment. Patients with second primary cancer, those with pleural effusion, those who had previously undergone surgery or radiation, and those who had received targeted treatment or consolidation immunotherapy were excluded from the study.

### Data collection and calculation of specific variables

The electronic medical records were used to collect information on the following variables: age, sex, performance status score, smoking status, pathology, staging, treatment method, tumor (T) stage, lymph node (N) stage, presence or absence of positron emission tomography/computed tomography scans, absolute neutrophil count (ANC), absolute lymphocyte count (ALC). ANC and ALC from all 188 patients were collected before radiotherapy and after completion of the full radiotherapy protocol.

The pre-treatment ANC was divided by the pre-treatment ALC to calculate the pre-treatment NLR. Similarly, the post-treatment ANC was divided by the post-treatment ALC to determine the post-treatment NLR. The ΔNLR was derived by subtracting the pre-treatment NLR from the post-treatment NLR.

The radiation therapy (RT) method, radiation dosage, GTV, clinical target volume (CTV), planning target volume (PTV), mean lung dose, and mean heart dose data were extracted from the radiotherapy plan.

### Radiotherapy

Three-dimensional conformal radiation therapy (3D-CRT), intensity-modulated radiation therapy (IMRT), and volumetric modulated arc therapy (VMAT) were the radiotherapy modalities used in the whole cohort, with a median dosage of 60 (range, 50–66) Gy.

### Statistical analysis

The following clinical outcomes were evaluated in this study: OS, local progression-free survival (LPFS), and distant metastasis-free survival (DMFS). OS was computed from the moment of diagnosis to the final follow-up or the time of death from any cause. LPFS was defined as the time from diagnosis to local progression or death from any cause. DMFS was defined as the time from diagnosis to distant metastasis or death from any cause.

Both univariate and multivariate Cox proportional hazards regression analyses were conducted to assess the association between potential prognostic variables and outcomes. Variables with *P* value < 0.1 in the univariate analysis were subsequently included in the multivariate regression analysis. A stepwise forward method was utilized to compute the results of the multivariate analysis. As a result of the regression coefficients, hazard ratios (HRs) were obtained. The assessment of OS, LPFS, and DMFS was conducted using Kaplan–Meier analysis. ΔNLR was divided into two groups for Kaplan–Meier analysis. Potential associations between post-treatment NLR, ΔNLR, and other prognostic markers were assessed by calculating Spearman’s rank correlation coefficient. The standard distribution test was conducted for the two groups of ΔNLR stratified by GTV. Normally distributed data were analyzed by t-test. Abnormally distributed data were analyzed by the non-parametric test. Patients received intensity-modulated radiation therapy was performed as a subgroup to analysis in order to overcome the hetero geneity of the study population. Statistical analysis was carried out using SPSS 26.0 software (IBM, Armonk, NY, USA). *P* < 0.05 was considered statistically significant.

## Results

### Patients’ characteristics

Totally, 188 patients were finally involved in this study. The median follow-up time was 46.5 months, with a maximum follow-up time of 112.8 months. Regrettably, 70.74% of patients passed away. Table 1 lists patients’ characteristics. Besides, 66.0% of patients underwent brain magnetic resonance imaging and PET examination for tumor staging. Stage IIIA (54.3%), IIIB (41.5%), or IIIC (4.2%) was used to classify all patients. Moreover, 42.6% of tumors were found in the left lung and 57.4% in the right lung.

**Table 1:** Patient characteristics.

Characteristic	Number of patients (N = 188)	%
Age (y)		
Median	61	
Range	30–88	
Gender		
Male	153	81.4
Female	35	18.6
ECOG-score		
0	105	55.9
1	83	44.1
Smoking history		
No	35	18.6
Histology		
Squamous cell carcinoma	115	61.2
Adenocarcinoma	69	36.7
Large cell carcinoma	4	2.1
T stage		
T1	26	13.8
T2	100	53.2
T3	24	12.8
T4	38	20.2
N stage		
N0	11	5.9
N1	12	6.4
N2	114	60.6
N3	51	27.1
Stage		
IIIA	102	54.3
IIIB	78	41.5
IIIC	8	4.2
PET staging		
Yes	124	66
No	64	34
Location		
Left hilum	6	3.2
Left upper lobe	43	22.9
Left lower lobe	31	16.5
Right hilum	14	7.4
Right upper lobe	56	29.8
Right middle lobe	19	10.1
Right lower lobe	19	10.1
Concurrent chemoradiotherapy		
Yes	76	40.4
No	112	59.6
RT technique		
3D-CRT	3	1.6
IMRT	154	81.9
VMAT	31	16.5
Delivered dose of RT (Gy)		
Median	60	
Range	50–66	
Average NLR level		
Pre-treatment NLR	2.58	
Post-treatment NLR	5.92	
Range of ΔNLR		
GTV ≤ 67.41 cm^3^ group	− 20.47	
GTV > 67.41 cm^3^ group	− 32.23	
ΔNLR ≤ 2.60 group	− 6.61–2.60	
ΔNLR > 2.60 group	2.61–25.98	

*ECOG* Eastern Cooperative Oncology Group, *T* tumor, *N* Node, *PET* positron emission tomography, *RT* radiation therapy, *3D-CRT* 3-dimensional conformal radiotherapy, *IMRT* intensity modulated radiation therapy, *VMAT* volumetric modulated arc therapy, *Gy* Gray, *y* years, *NLR* neutrophil-to-lymphocyte ratio, *ΔNLR* neutrophil-to-lymphocyte ratio change.

### The relationship between variables

**Figure 1:**
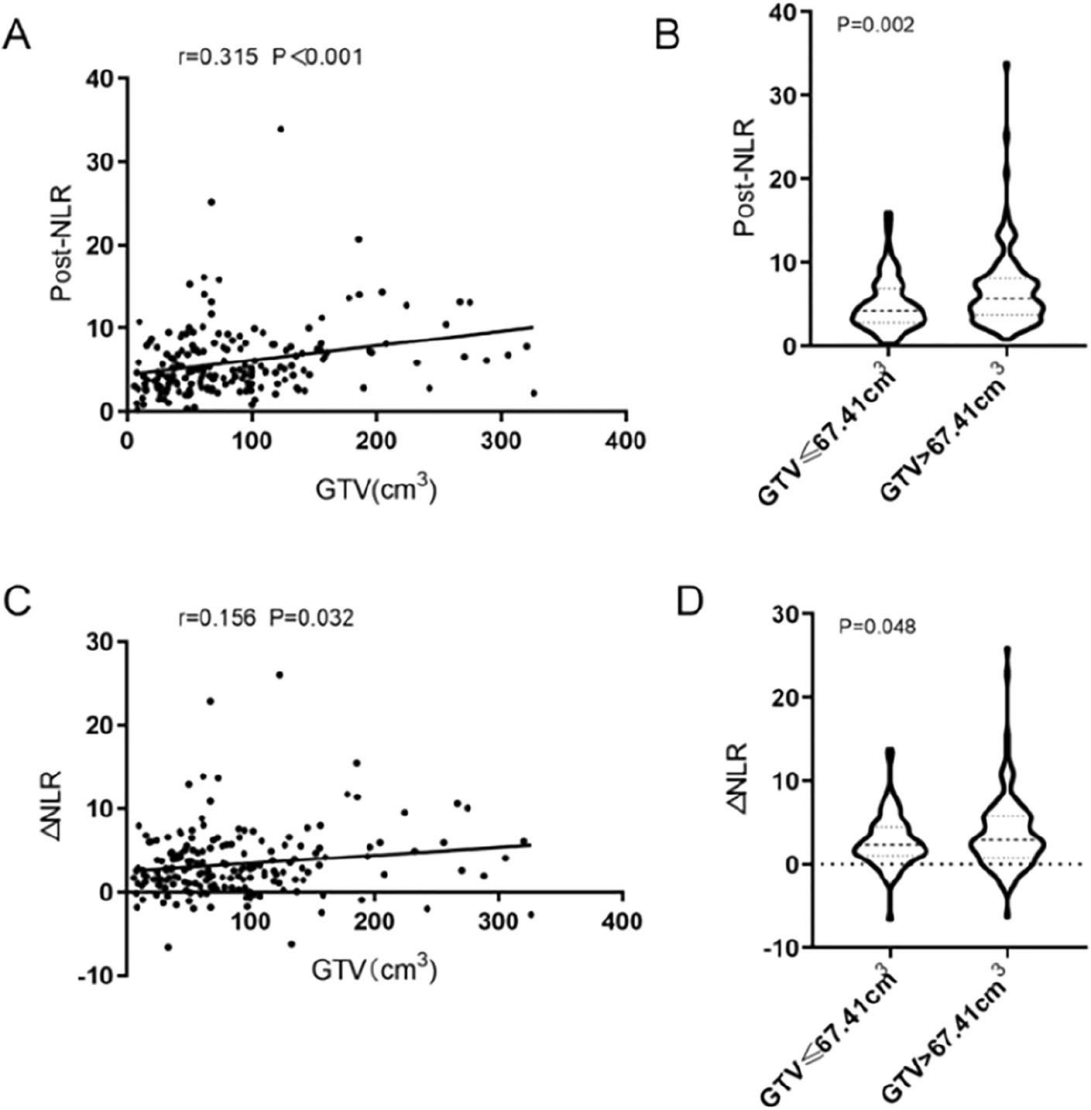
The relationship between post-treatment NLR, ΔNLR and GTV (**A**) Spearman’s correlation coefficients for post–treatment NLR and GTV, (**B**) After grouping post–treatment NLR according to the median of GTV, (**C**) Spearman’s correlation coefficients for ΔNLR and GTV, (**D**) After grouping ΔNLR according to the median of GTV.

The median pre-treatment NLR was 2.10 (range, 0.09–10.62). The median post-treatment NLR was 4.80 (range, 0.22–33.91). The median ΔNLR was 2.60 (range, − 6.61–25.98). In the whole cohort, the median GTV was 67.41 (range, 5.39–326.0) cm^3^. Post-treatment NLR and GTV were linearly and positively associated together, according to the Spearman rank correlation (*r* = 0.315, *P* < 0.001) (Fig. 1A). After grouping post-treatment NLR according to the median GTV (67.41 cm^3^), the average post-treatment NLR of 4.99 (3.16 ± 0.33) in the GTV ≤ 67.41 cm^3^ group was significantly lower than that (6.85, 4.94 ± 0.51) in the GTV > 67.41 cm^3^ group (*P* = 0.002, Fig. 1B). Furthermore, ΔNLR and GTV exhibited to have a linear positive association together, according to the Spearman rank correlation (*r* = 0.156, *P* = 0.032, Fig. 1C). After grouping △NLR according to the median GTV (67.41 cm^3^), the mean ΔNLR in the GTV ≤ 67.41 cm^3^ group was 2.79 (3.01 ± 0.31), which was significantly lower than that in the GTV > 67.41 cm^3^ group (3.89, 4.79 ± 0.49) (*P* = 0.048, Fig. 1D).

### The association of variables with prognosis

**Figure 2:**
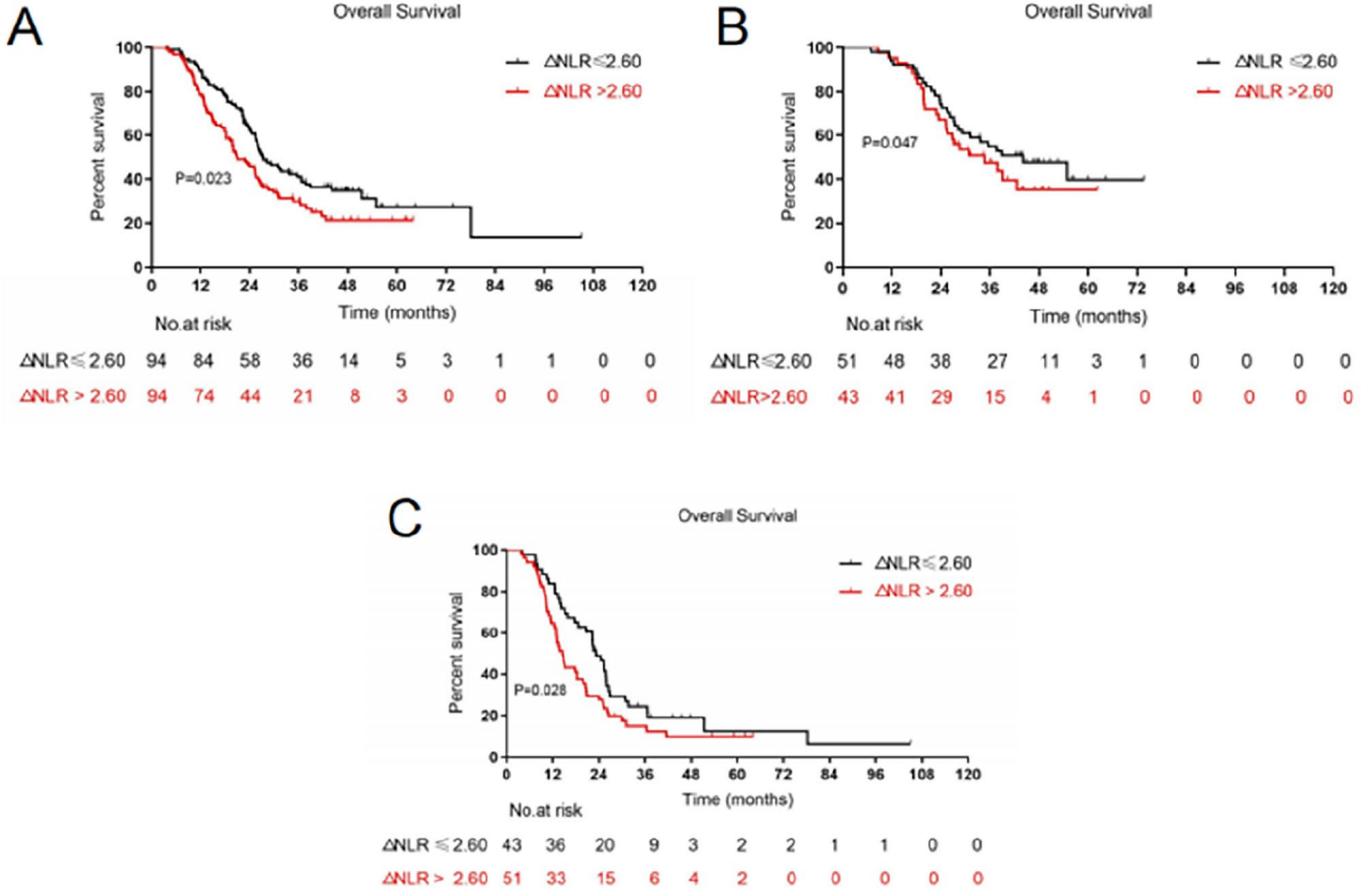
Kaplan–Meier curves for overall survival by dichotomy (**A**) in the entire cohort, (**B**) in GTV ≤ 67.41 cm^3^ group, and (**C**) in GTV > 67.41 cm^3^ group. Patients were stratified by ranges of ΔNLR.

**Figure 3:**
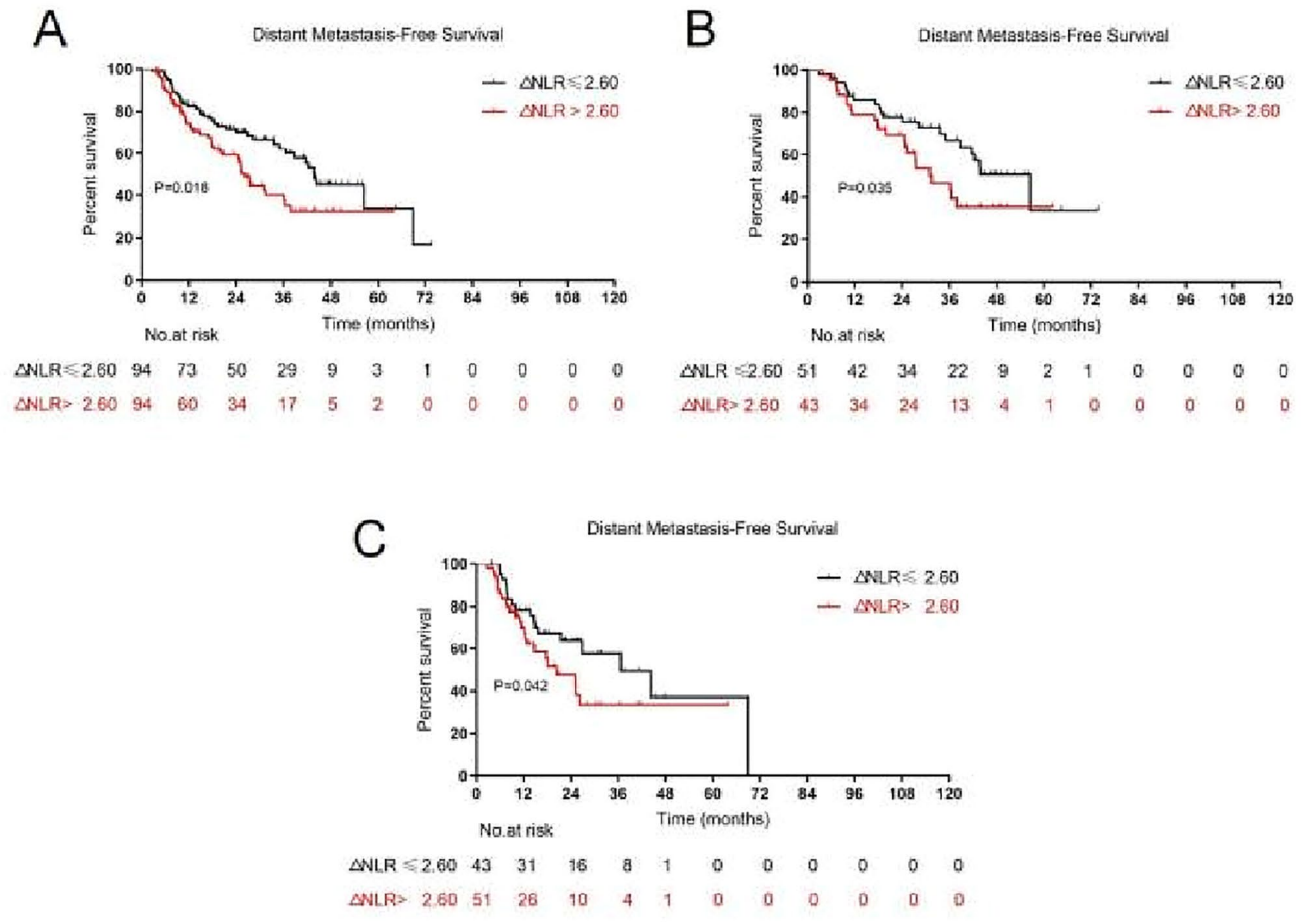
Kaplan–Meier curves for distant metastasis-free survival by dichotomy (**A**) in the entire cohort, (**B**) in GTV ≤ 67.41 cm^3^ group, and (**C**) in GTV > 67.41 cm^3^ group. Patients were stratified by ranges of the ΔNLR.

With median durations of 25.3, 20.4, and 36.1 months, the 5-year OS, LPFS, and DMFS rates were 24.4, 28.3, and 31.8%, respectively. A larger GTV was strongly correlated with OS, LPFS, and DMFS based on the results of univariate analysis. OS and DMFS were significantly correlated with the increased post-treatment NLR and ΔNLR. However, pre-treatment NLR was found irrelevant to OS, LPFS, or DMFS (Table 2). The results of multivariate analysis indicated that a larger GTV was associated with worse OS, LPFS, and DMFS, while a greater ΔNLR indicated a significant correlation with worse OS and DMFS. It is also noteworthy that multivariate analysis did not reveal any associations between post-treatment NLR and OS or DMFS (Table 3). For further research, Kaplan–Meier analysis was used to dichotomize ΔNLR; a greater ΔNLR was linked to worse OS (*P* = 0.023, Fig. 2A) and DMFS (*P* = 0.018, Fig. 3A). Median values of OS and DMFS in the low ΔNLR group (27.2 months, 95% confidence interval (CI): 22.2–32.2 and 43.8 months, 95%CI 34.7–52.9 months, respectively) were higher than those in the high post-treatment NLR group (20.7 months, 95%CI 15.7–25.8 and 26.2 months, 95% CI 23.2–29.2 months, respectively).

**Table 2:** Univariable analysis of clinical and dosimetric variables with outcomes.

Variables	OS		LPFS		DMFS	
	HR (95%CI)	*P*	HR (95%CI)	*P*	HR (95%CI)	*P*
Age (years)	1.002 (0.982, 1.023)	0.842	1.009 (0.988, 1.031)	0.379	1.001 (0.977, 1.026)	0.93
Sex	1.330 (0.847, 2.086)	0.215	1.426 (0.880, 2.312)	1.15	1.144 (0.870, 1.503)	0.335
ECOG score	0.986 (0.959, 1.013)	0.301	0.979 (0.959, 1.018)	0.421	1.009 (0.975, 1.044)	0.663
Smoking history	0.735 (0.468, 1.153)	0.18	0.542 (0.323, 0.910)	0.02	1.159 (0.909, 1.477)	0.235
Histology	1.188 (0.847, 1.665)	0.319	1.256 (0.879, 1.793)	0.211	0.881 (0.584, 1.328)	0.545
Tumor location	1.178 (0.835, 1.661)	0.351	1.235 (0.855, 1.783)	0.26	0.913 (0.735, 1.135)	0.414
T stage	1.221 (1.025, 1.453)	0.025	1.215 (1.001, 1.474)	0.048	1.054 (0.847, 1.132)	0.636
N stage	0.968 (0.768, 1.220)	0.782	1.119 (0.862, 1.451)	0.398	1.211 (0.887, 1.653)	0.229
Staging with PET	1.179 (0.817, 1.692)	0.384	1.178 (0.798, 1.741)	0.41	1.039 (0.666, 1.620)	0.867
Total RT dose	1.007 (0.970, 1.046)	0.701	1.019 (0.978, 1.061)	0.374	0.998 (0.951, 1.046)	0.92
Radiation technology	1.101 (0.729, 1.660)	0.648	0.839 (0.536, 1.314)	0.444	1.340 (0.810, 2.217)	0.255
GTV (cm^3^)	1.006 (1.003, 1.008)	< 0.001	1.004 (1.001, 1.006)	0.006	1.004 (1.001, 1.007)	0.004
CTV (cm^3^)	1.002 (1.001, 1.003)	< 0.001	1.002 (1.000, 1.003)	0.013	1.002 (1.001, 1.004)	0.001
PTV (cm^3^)	1.001 (1.000, 1.002)	0.004	1.001 (1.000, 1.002)	0.049	1.002 (1.001, 1.002)	0.001
MLD	1.000 (1.000, 1.001)	0.693	1.001 (1.000, 1.002)	0.028	1.001 (1.000, 1.002)	0.13
MHD	1.000 (1.000, 1.000)	0.262	1.000 (1.000, 1.000)	0.219	1.000 (1.000, 1.001)	0.086
Pre-treatment ANC	1.029 (0.967, 1.094)	0.372	0.980 (0.909, 1.058)	0.608	1.008 (0.931, 1.091)	0.845
Post-treatment ANC	1.204 (1.075, 1.347)	0.001	1.000 (1.000, 1.000)	0.268	1.094 (1.940, 1.274)	0.244
Pre-treatment ALC	1.056 (0.804, 1.388)	0.695	0.997 (0.731, 1.361)	0.987	0.960 (0.676, 1.365)	0.821
Post-treatment ALC	0.652 (0.343, 1.240)	0.192	1.365 (0.719, 2.589)	0.342	0.451 (0.197, 1.031)	0.059
Pre-treatment NLR	1.037 (0.941, 1.142)	0.464	0.974 (0.870, 1.091)	0.654	1.014 (0.899, 1.144)	0.821
Post-treatment NLR	1.074 (1.032, 1.119)	0.001	0.653 (0.344, 1.241)	0.194	1.062 (1.012, 1.115)	0.015
ΔNLR	1.072 (1.027, 1.119)	0.002	1.021 (0.973, 1.070)	0.401	1.062 (1.010, 1.118)	0.02

*ECOG* Eastern Cooperative Oncology Group, *T* tumor, *N* Node, *PET* positron emission tomography, *RT* radiation therapy, *GTV* gross tumor volume, *CTV* clinical target volume, *PTV* planning target volume, *MLD* mean lung dose, *MHD* mean heart dose, *ANC* absolute neutrophil count, *ALC* absolute lymphocyte count, *NLR* neutrophil-to-lymphocyte ratio, *ΔNLR* neutrophil-to-lymphocyte ratio change, *HR* hazard ratio, *OS* overall survival, *LPFS* local progression-free survival, *DMFS* distant metastasis-free survival.

**Table 3:** Multivariate analysis of clinical and dosimetric variables with outcomes.

Variables	OS		LPFS		DMFS	
	HR (95%CI)	*P*	HR (95%CI)	*P*	HR (95%CI)	*P*
Smoking history			0.548 (0.326, 0.924)	0.024		
T stage		0.496		0.235		
GTV (cm^3^)	1.005 (1.003, 1.007)	< 0.001	1.003 (1.000, 1.006)	0.041	1.004 (1.001, 1.007)	0.011
CTV (cm^3^)		0.525		0.702		0.475
PTV (cm^3^)		0.581		0.455		0.454
MLD			1.001 (1.000, 1.002)	0.049		
MHD						0.212
Post-treatment ANC		0.213				
Post-treatment ALC						0.326
Post-treatment NLR		0.849				0.877
ΔNLR	1.054 (1.008, 1.101)	0.020			1.054 (1.001, 1.110)	0.046

*T* tumor, *GTV* gross tumor volume, *CTV* clinical target volume, *PTV* planning target volume, *MLD* mean lung dose, *MHD* mean heart dose, *ANC* absolute neutrophil count, *ALC* absolute lymphocyte count, *NLR* neutrophil-to-lymphocyte ratio, *ΔNLR* neutrophil-to-lymphocyte ratio change, *HR* hazard ratio, *OS* overall survival, *LPFS* local progression-free survival, *DMFS* distant metastasis-free survival.

In the univariate analysis and Kaplan–Meier analysis, GTV was utilized as a stratification factor since the association between ΔNLR and GTV could affect the prognostic function of ΔNLR. After using GTV as a stratification factor in the univariate analysis, a significant association was identified between ΔNLR and OS (HR = 1.058, *P* = 0.007). ΔNLR was correlated with OS in the GTV ≤ 67.41 cm^3^ group (*P* = 0.047, Fig. 2B) and the GTV > 67.41 cm^3^ group (*P* = 0.028, Fig. 2C) when ΔNLR was dichotomized on the Kaplan–Meier analysis. After incorporating GTV as a stratification factor in the univariate analysis, the association between ΔNLR and DMFS remained significant (HR = 1.057, *P* = 0.031). When ΔNLR was dichotomized for the Kaplan–Meier analysis, ΔNLR was correlated with DMFS in both the GTV ≤ 67.41 cm^3^ (*P* = 0.035, Fig. 3B) and GTV > 67.41 cm^3^ groups (*P* = 0.042, Fig. 3C).

In the subgroup analysis of patients received intensity-modulated radiation therapy, The results of univariate analysis (S Table 1) and multivariate analysis (S Table 2) were similar to those of the whole population.

## Discussion

In the present research, the determinants of post-treatment NLR and ΔNLR in patients with thoracic radiotherapy-treated LA-NSCLC were investigated. After incorporating the covariates, the prognostic significance of pre-treatment NLR, post-treatment NLR, and ΔNLR was subsequently evaluated. It was found that GTV was associated with post-treatment NLR and ΔNLR. In the multivariate analysis, ΔNLR was regarded as an independent predictor of OS and DMFS after GTV addition. Besides, there was no statistically significant difference between pre-treatment NLR, post-treatment NLR, and prognosis.

Inflammation and the growth and metastasis of tumors are tightly connected^12–14^, and it is more important to explore the impact of inflammatory status on prognosis with the widely application of immunotherapy in lung cancer. NLR has been widely studied as one of the important markers of inflammation. However, the predictive value of NLR for the prognosis of LA-NSCLC needs further exploration especially in the era of immunotherapy. In NSCLC patients with stage II and stage III who received radical intensity-modulated radiation therapy, prior research has shown that a lower baseline NLR indicates higher rates of PFS and OS^15^. Conversely, baseline NLR was confirmed to have no association with OS in III NSCLC patients, of whom 115 (35%) patients underwent surgical resection, followed by chemotherapy or chemoradiotherapy, and the remaining 217 (65%) patients received synchronous CRT^16^. Furthermore, baseline NLR was not associated with prognosis in stage IIB-IIIC NSCLC patients receiving radical radiotherapy^17^. Similarly, pre-treatment NLR and OS, LPFS, and DMFS were not correlated together in the present research (*P* = 0.464, 0.654, and 0.821, respectively). This result was not surprising because the immune state is dynamic, which can be changed by radiotherapy or chemotherapy. It is broadly accepted that radiotherapy is one of the main treatment methods for LA-NSCLC, which can not only directly kill tumor cells, but also impact the body's immune system^18^. Relevant studies have revealed that radiation can reduce the number of peripheral blood immune cells, especially lymphocytes. Lymphocytes are more sensitive to radiation, in which 50% lethal dose is as low as 2 Gy^19^, and 0.5 Gy can cause DNA damage^20,21^. Thus, the number of lymphocytes can be significantly reduced by radiotherapy^22,23^. Therefore, post-treatment NLR and ΔNLR may provide more information on the prognosis than pre-treatment NLR, and further determining its predictive value is more meaningful. Post-treatment NLR and ΔNLR were previously found as independent predictors of poor survival in NSCLC patients following radical radiation therapy^9^. Besides, NLR at four months after radiotherapy was also associated with OS^6^. Consistently, the data of stage III NSCLC patients treated with radiation therapy and chemotherapy were related to the post-treatment NLR^7^. High postoperative NLR was an independent unfavorable prognostic factor in patients with LA-NSCLC who were treated with surgery after chemoradiotherapy with or without postoperative adjuvant chemotherapy^24^. In patients receiving concomitant chemoradiotherapy, ΔNLR was reported as a standalone predictor of OS and PFS^10^. Further research revealed that PFS and OS in Kaplan–Meier survival analysis were correlated with post-treatment NLR and ΔNLR. Additionally, the results of multivariate analysis indicated that post-treatment NLR could only be utilized as an independent predictor of PFS, whereas ΔNLR was noted as an independent predictor of PFS and OS^25^.

To some extent, the abovementioned studies suggest that the post-treatment NLR and the ΔNLR after treatment are associated with prognosis. It is worth noting that the majority of the prognostic indicators used in the aforementioned studies are OS and PFS. The body’s immune status may be highly correlated with DMFS. Furthermore, previous investigations did not consider putative post-treatment NLR and ΔNLR determinants (e.g., GTV size), which may affect the outcomes. In earlier trials, a significant baseline GTV was linked to poorer OS and PFS^26–28^. Hence, it is necessary to explore the predictive significance of post-treatment NLR and ΔNLR excluding the influence of GTV and add DMFS as a prognostic indicator. This study was revealed that GTV was a standalone unfavorable prognostic factor for OS, LPFS, and DMFS; of note, a larger GTV was associated with higher values of post-treatment NLR and △NLR. This could be attributed to the more extensive radiation range of the larger GTV group. A more extensive radiation range signifies more exposure of immune cells and a more significant reduction in the number of lymphocytes. Therefore, the prognostic significance of pre-treatment NLR, post-treatment NLR and ΔNLR after GTV addition remains to be further verified. It is noteworthy that although the univariate analysis showed that post-treatment NLR was associated with OS and DMFS, no statistically significant difference was found in the results of the multivariate analysis. Additionally, ΔNLR remains a separate prognostic factor for OS and DMFS when GTV is taken into account in the multivariate analysis. Furthermore, ΔNLR exhibited to have no association with LPFS. The conjecture is that GTV is more closely correlated with local control, and the severity of compromised immune system is associated with the distant metastasis of the tumor. The Kaplan–Meier survival analysis also supported these results. These results indicate that ΔNLR is more closely correlated with prognosis, especially it discovered the connection of ΔNLR and DMFS which different from other studies. This result suggests that the immune status of the body is crucial for controlling of the systemic tumors, and it is very meaningful especially in the era of immunotherapy. It is well known that although immune maintenance therapy after radiochemotherapy has improved the efficacy, The 12-, 24- and 36- month OS rates with durvalumab were 83.1, 66.3, and 57.0%^1–3^, and there is significant population heterogeneity. Therefore, it is necessary to further stratify and develop more accurate treatment methods. The results of this study .indicating that ΔNLR may serve as a stratified indicator to assist clinical differentiation of beneficiary populations and the development of more accurate treatment decisions.

Because of the significant heterogeneity of the population and the majority of people receiving intensity-modulated radiation therapy (81.9%), we performed subgroup analysis. However, the results indicate that ΔNLR is also correlated with OS and DMFS whicn similar to those of the whole population.

Although the present research established the association between ΔNLR and GTV, and it also confirmed the prognostic value of ΔNLR in stage LA-NSCLC, there are some limitations that should be pointed out. Firstly, this research inherently involved selection bias owing to its retrospective design. Secondly, NLR might be influenced by potential confounding factors (e.g., possible infection and chemotherapy regimen). Thirdly, the potential variations in salvage therapies may have inadvertently influenced the outcomes. Finally, this was a single-center study, and findings should be verified externally in future research.

## Conclusions

In conclusion, post-treatment NLR and ΔNLR were found to be associated with GTV. The impact of ΔNLR on survival and metastasis remained noticeable when GTV was analyzed. Furthermore, there was no statistically significant difference between pre-treatment NLR, post-treatment NLR, and prognosis. Further research should be conducted to clarify the prognostic significance of ΔNLR.

### Supplementary Information

Supplementary Information.

## Data Availability

The datasets generated during and/or analysed during the current study are available from the corresponding author on reasonable request.

## References

[1] Antonia SJ (2017). Durvalumab after chemoradiotherapy in stage III non-small-cell lung cancer. N. Engl. J. Med..

[2] Gray JE (2020). Three-year overall survival with durvalumab after chemoradiotherapy in stage III NSCLC-update from PACIFIC. J. Thorac. Oncol..

[3] Faivre-Finn C (2021). Four-year survival with durvalumab after chemoradiotherapy in stage III NSCLC-an update from the PACIFIC trial. J. Thorac. Oncol..

[4] Ohri N (2021). Who benefits the most from adjuvant durvalumab after chemoradiotherapy for non-small cell lung cancer? An exploratory analysis. Pract. Radiat. Oncol..

[5] Scilla KA (2017). Neutrophil-lymphocyte ratio is a prognostic marker in patients with locally advanced (Stage IIIA and IIIB) non-small cell lung cancer treated with combined modality therapy. Oncologist.

[6] Thor M (2020). Are unsatisfactory outcomes after concurrent chemoradiotherapy for locally advanced non-small cell lung cancer due to treatment-related immunosuppression?. Radiother. Oncol..

[7] Palomar-Abril V (2020). Dynamic evaluation of neutrophil-to-lymphocyte ratio as prognostic factor in stage III non-small cell lung cancer treated with chemoradiotherapy. Clin. Transl. Oncol..

[8] Łochowski M (2019). Prognostic value of neutrophil-to-lymphocyte, platelet-to-lymphocyte and lymphocyte-to-monocyte ratio ratios in patients operated on due to non-small cell lung cancer. J. Thorac. Dis..

[9] Punjabi A (2021). Neutrophile lymphocyte ratio and absolute lymphocyte count as prognostic markers in patients treated with curative-intent radiotherapy for non-small cell lung cancer. Clin. Oncol..

[10] Guo M (2019). Prognostic value of delta inflammatory biomarker-based nomograms in patients with inoperable locally advanced NSCLC. Int. Immunopharmacol..

[11] Kwinta M (2020). The prognostic value of volumetric changes of the primary tumor measured on Cone Beam-CT during radiotherapy for concurrent chemoradiation in NSCLC patients. Radiother. Oncol..

[12] Shadab A (2023). Divergent functions of NLRP3 inflammasomes in cancer: A review. Cell Commun. Signal..

[13] Jiao ZY, Zhang J (2023). Interplay between inflammasomes and PD-1/PD-L1 and their implications in cancer immunotherapy. Carcinogenesis.

[14] Li X (2021). The immunological and metabolic landscape in primary and metastatic liver cancer. Nat. Rev. Cancer.

[15] Li Y (2023). Higher aorta dose increased neutrophil-to-lymphocyte ratio resulting in poorer outcomes in stage II–III non-small cell lung cancer. Thorac. Cancer..

[16] Tong YS, Tan J, Zhou XL, Song YQ, Song YJ (2017). Systemic immune-inflammation index predicting chemoradiation resistance and poor outcome in patients with stage III non-small cell lung cancer. J. Transl. Med..

[17] Ozkan EE, Kaymak Cerkesli ZA, Erdogan M (2020). Predictive value of immune-inflammation indices in metabolic response and outcome after curative radiotherapy in patients with non-small cell lung cancer. Clin. Respir. J..

[18] Yin X (2021). Is a higher estimated dose of radiation to immune cells predictive of survival in patients with locally advanced non-small cell lung cancer treated with thoracic radiotherapy?. Radiother. Oncol..

[19] Nakamura N, Kusunoki Y, Akiyama M (1990). Radiosensitivity of CD4 or CD8 positive human T-lymphocytes by an in vitro colony formation assay. Radiat. Res..

[20] Heylmann D, Rödel F, Kindler T, Kaina B (2014). Radiation sensitivity of human and murine peripheral blood lymphocytes, stem and progenitor cells. Biochim. Biophys. Acta (BBA) Rev. Cancer.

[21] Sellins KS, Cohen JJ (1987). Gene induction by gamma-irradiation leads to DNA fragmentation in lymphocytes. J. Immunol..

[22] Kobzeva I (2023). Effect of radiation therapy on composition of lymphocyte populations in patients with primary breast cancer. J. Pers. Med..

[23] Paganetti H (2023). A review on lymphocyte radiosensitivity and its impact on radiotherapy. Front. Oncol..

[24] Tsudaka S (2021). Prognostic significance of neutrophil-to-lymphocyte ratio in locally advanced non-small-cell lung cancer treated with trimodality therapy. Ann. Surg. Oncol..

[25] Wang D (2020). The post-treatment neutrophil-to-lymphocyte ratio and changes in this ratio predict survival after treatment of stage III non-small-cell lung cancer with conventionally fractionated radiotherapy. Future Oncol..

[26] Nygard L (2018). A competing risk model of first failure site after definitive chemoradiation therapy for locally advanced non-small cell lung cancer. J. Thorac. Oncol..

[27] Edith MT (2018). Concurrent daily cisplatin and high-dose radiation therapy in patients with stage III non-small cell lung cancer. Int. J. Radiat. Oncol. Biol. Phys..

[28] Zhou R, Xu T (2018). Radiation dose, local disease progression, and overall survival in patients with inoperable non-small cell lung cancer after concurrent chemoradiation therapy. Int. J. Radiat. Oncol. Biol. Phys..

