# Supplementary material for: Prognostic value of neutrophil-to-lymphocyte ratio change in patients with locally advanced non-small cell lung cancer treated with thoracic radiotherapy
Source: Sci Rep. 2024 May 25;14:11984. doi: 10.1038/s41598-024-62662-3 (PMC11127913; doi:10.1038/s41598-024-62662-3)
Supplement: Supplementary file 1 — Supplementary Information. [file 41598_2024_62662_MOESM1_ESM.docx]

**S Table 1** Univariable analysis of clinical and dosimetric variables with outcomes in intensity-modulated radiation therapy subgroup

OS LPFS DMFS

Variables

HR（95%CI） P HR（95%CI） P HR（95%CI） P

Age (years) 0.998(0.976,1.020) 0.829 1.005(0.982,1.029) 0.665 0.995(0.967,1.023) 0.717

Sex 1.208(0.749,1.949) 0.438 1.386(0.828,2.319) 0.215 1.287(0.711,2.328) 0.405

ECOG score 0.990(0.959,1.022) 0.537 0.996(0.962,1.031) 0.805 1.017(0.977,1.059) 0.400

Smoking history 0.900(0.553,1.466) 0.673 0.666(0.381,1.162) 0.152 1.275(0.725,2.240) 0.399

Histology 1.303(0.880,1.930) 0.187 1.213(0.808,1.820) 0.352 0.970(0.599,1.569) 0.900

Tumor location 1.019(0.692,1.503) 0.923 1.221(0.811,1.839) 0.339 0.716(0.432,1.187) 0.196

T stage 1.205(0.993,1.462) 0.058 1.265(1.023,1.564) 0.030 1.024(0.799,1.313) 0.852

N stage 1.003(0.763,1.320) 0.980 1.296(0.938,1.792) 0.116 1.143(0.791,1.650) 0.477

Staging with PET 1.207(0.800,1.822) 0.369 1.204(0.777,1.864) 0.406 1.139(0.683,1.900) 0.618

Total RT dose 1.018(0.977,1.060) 0.408 1.022(0.977,1.070) 0.345 1.000(0.948,1.056) 0.992

GTV (cm^3^) 1.005(1.003,1.008) ＜0.001 1.003(1.001,1.006) 0.017 1.005(1.002,1.008) 0.004

MLD 1.000(1.000,1.001) 0.448 1.001(1.000,1.002) 0.012 1.001(1.000,1.002) 0.188

MHD 1.000(1.000,1.000) 0.377 1.000(1.000,1.000) 0.273 1.000(1.000,1.001) 0.198

Pre-treatment ANC 1.046(0.957,1.143) 0.325 0.968(0.873,1.073) 0.535 1.030(0.922,1.150) 0.598

Post-treatment ANC 1.159(0.906,1.474) 0.234 1.199(1.042,1.379) 0.011 1.196(0.887,1.410) 0.203

Pre-treatment ALC 1.124(0.833,1.515) 0.445 1.147(0.814,1.617) 0.433 0.988(0.666,1.466) 0.952

Post-treatment ALC 0.660(0.315,1.384) 0.272 1.647(0.783,3.462) 0.188 0.661(0.261,1.673) 0.382

Pre-treatment NLR 1.025(0.905,1.160) 0.702 0.938(0.810,1.085) 0.390 1.025(0.881,1.192) 0.749

Post-treatment NLR 1.073(1.024,1.124) 0.003 1.007(0.952,1.066) 0.798 1.075(1.017,1.136) 0.011

ΔNLR 1.076(1.024,1.130) 0.004 1.018(0.963,1.077) 0.523 1.076(1.016,1.140) 0.012

*Abbreviations:* ECOG: Eastern Cooperative Oncology Group; T = tumor; N = Node; PET = positron emission tomography; RT = radiation therapy; GTV= gross tumor volume; MLD = mean lung dose; MHD = mean heart dose; ANC = absolute neutrophil count; ALC = absolute lymphocyte count; NLR = neutrophil-to-lymphocyte ratio; ΔNLR = neutrophil-to-lymphocyte ratio change; HR = hazard ratio; OS = overall survival; LPFS = local progression-free survival; DMFS = distant metastasis-free survival.

**S Table 2** Multivariate analysis of clinical and dosimetric variables with outcomes in intensity-modulated radiation therapy subgroup

OS LPFS DMFS

Variables

HR（95%CI） P HR（95%CI） P HR（95%CI） P

T stage 0.561 0.277

GTV (cm^3^) 1.005(1.002,1.007) ＜0.001 1.003(1.000,1.006) 0.049 1.004(1.001,1.007) 0.009

MLD 1.001(1.000,1.002) 0.013

Post-treatment ANC 1.173(1.013,1.358) 0.032

Post-treatment NLR 0.602 0.820

ΔNLR 1.060(1.007,1.116) 0.027 1.066(1.005,1.131) 0.034

*Abbreviations:* T = tumor; GTV= gross tumor volume; MLD = mean lung dose; ANC = absolute neutrophil count; NLR = neutrophil-to-lymphocyte ratio; ΔNLR = neutrophil-to-lymphocyte ratio change; HR = hazard ratio; OS = overall survival; LPFS = local progression-free survival; DMFS = distant metastasis-free survival.
